# Short-Duration Maximal and Long-Duration Submaximal Effort Forearm Exercise Achieve Elevations in Serum Brain-Derived Neurotrophic Factor

**DOI:** 10.3389/fphys.2017.00746

**Published:** 2017-10-06

**Authors:** Jeremy J. Walsh, Robert F. Bentley, Brendon J. Gurd, Michael E. Tschakovsky

**Affiliations:** ^1^Human Vascular Control Lab, School of Kinesiology and Health Studies, Queen's University, Kingston, ON, Canada; ^2^Queen's Muscle Physiology Lab, School of Kinesiology and Health Studies, Queen's University, Kingston, ON, Canada

**Keywords:** thrombocytosis, platelets, neurotrophin, BDNF dose, exercise intensity, handgrip exercise

## Abstract

Brain-derived neurotrophic factor (BDNF) is a major orchestrator of exercise-induced brain plasticity and circulating (peripheral) BDNF may have central effects. Approximately 99% of circulating BDNF is platelet-bound, and at rest ~30% of circulating platelets are stored in the spleen. Interestingly, forearm handgrip exercise significantly elevates sympathetic outflow and has been shown to induce splenic constriction, suggesting that small muscle mass exercise could stand as a viable strategy for increasing circulating BDNF; however, the BDNF response to handgrip exercise is currently unknown.

**Purpose:** This study examined BDNF and platelet responses to short-duration maximal (ME) and prolonged submaximal (SE) effort handgrip exercise.

**Methods:** Healthy males (*n* = 18; 21.4 ± 2.1 years, BMI 25.0 ± 1.0 kg/m^2^) performed 10 min of ME and 30 min of SE. Blood was sampled for the determination of serum BDNF and platelet count at rest and during the last minute of exercise.

**Results:** Compared to rest, serum BDNF significantly increased during ME (21.2%) and SE (11.2%), which displayed a non-significant trend toward an intensity-dependent response. Platelets increased in an intensity-dependent fashion compared to rest with an 8.0% increase during ME and 3.1% during SE, and these responses were significantly correlated with diastolic blood pressure responses to handgrip exercise. Further, the amount of BDNF per platelet significantly increased compared to rest during ME (13.4%) and SE (8.7%).

**Conclusions:** Handgrip exercise evokes significant increases in serum BDNF and platelets, implicating splenic constriction as a key mechanism and confirming efficacy of this exercise model for elevating circulating BDNF.

## Introduction

Brain-derived neurotrophic factor (BDNF) is chiefly recognized for its role in orchestrating activity-dependent brain plasticity (Cotman et al., 2007). At the central nervous system (CNS) level, BDNF is critical for learning and memory processes, and is a key regulator of neuronal growth and integrity (Phillips et al., 2014). Interestingly, there is an association between CNS and circulating levels of BDNF, suggesting that peripheral levels may be important for CNS function (Angelucci et al., 2011). In support of this are findings from studies on depression and aging. In a murine model of depression it was demonstrated that chronic peripheral infusion of BDNF resulted in antidepressant behavioral and neuronal adaptations (Schmidt and Duman, 2010). Human studies reveal that individuals with depression have significantly lower circulating BDNF compared to healthy controls (Brunoni et al., 2008). Treatment with antidepressant drugs significantly elevates circulating BDNF and these changes are significantly correlated with improved depression scores (Brunoni et al., 2008). Finally, the age-associated decline in circulating BDNF (Lommatzsch et al., 2005; Ziegenhorn et al., 2007) is thought to mediate hippocampal shrinkage and worsening of spatial memory (Erickson et al., 2010). Given this evidence, exploration into means by which circulating BDNF can be augmented has important implications for brain health in both clinical and healthy populations, alike.

Physical exercise is one particularly effective strategy for increasing circulating levels of BDNF (Knaepen et al., 2010; Szuhany et al., 2015). It has repeatedly been demonstrated that an acute bout of aerobic exercise transiently increases both serum and plasma BDNF in an intensity-dependent manner (Knaepen et al., 2010; Szuhany et al., 2015). While the means by which exercise evokes this transient BDNF response is poorly understood, the current dogma accepts the brain as the primary source of plasma BDNF at rest and during exercise (Rasmussen et al., 2009; Seifert et al., 2010). However, the plasma BDNF accounts for only 1% of total circulating BDNF (Radka et al., 1996). Given that previous studies have observed increases in serum BDNF >30% following aerobic exercise (Ferris et al., 2007; Cho et al., 2012; Gilder et al., 2014), and that serum has ~200 times greater BDNF content than plasma (Rosenfeld et al., 1995), serum responses to exercise are not explained by a cerebral source. As such, consideration must be given to additional sources of BDNF during exercise, especially when considered within the context of developing interventions to significantly elevate circulating BDNF.

The spleen is likely a significant contributor to elevated BDNF with exercise via the release of platelets (Matthews et al., 2009; Cho et al., 2012) and the magnitude of this release appears to be exercise intensity-dependent (exercise-induced thrombocytosis; Chamberlain et al., 1990; Hulmi et al., 2010). Given that nearly 99% of blood-borne BDNF is platelet-bound (Radka et al., 1996), the addition of splenic platelets to the blood via thrombocytosis could account for a majority of the increased BDNF observed in response to exercise. Importantly, this increase in platelet-bound BDNF is not an inert pool; rather, this BDNF should be considered bioavailable as platelets release BDNF under conditions of increased shear stress (Fujimura et al., 2002), which is present in active muscle (Kellawan and Tschakovsky, 2014) and the cerebral vasculature during exercise (Jørgensen et al., 1992). Further, the increase in circulating catecholamines that accompanies exercise increases platelet activation, thereby potentiating the release of BDNF with exercise (Ahmadizad et al., 2006; Matthews et al., 2009).

In support of the dynamic contribution of platelets to bioavailable BDNF, platelet release of BDNF is significantly blunted in individuals with depression and this is evidenced by significantly lower levels of serum BDNF in this population despite having similar whole-blood levels to healthy controls (Karege et al., 2005). As such, platelets should be considered an important contributor of bioavailable BDNF and any exercise modality that can increase circulating platelets may have the potential to improve brain health. Traditional exercise modalities (i.e., aerobic or resistance exercise) may be a potent stimulus for augmenting platelets and BDNF (Matthews et al., 2009; Cho et al., 2012); however, the large muscle mass recruited with such exercise may not be requisite to evoke a significant platelet and serum BDNF response.

A closer look at the mechanism of thrombocytosis supports the potential for forearm handgrip exercise as a modality to achieve increases in serum BDNF. Thrombocytosis is the result of splenic constriction, caused by sympathetic stimulation of α-adrenergic receptors within the walls of the spleen (Chamberlain et al., 1990; Bakovic et al., 2013). Norepinephrine infusion causes splenic constriction and increases circulating platelets in a dose-dependent manner (Thoenen et al., 1964; Sloand et al., 1988). Similarly, low-dose infusion of epinephrine significantly increases circulating platelets due to splenic constriction in humans (Bakovic et al., 2013). Stewart et al. (2003) found that following exercise spleen volume was inversely correlated with levels of circulating catecholamines. Therefore, an exercise modality that evokes sufficient sympathetic outflow could stimulate exercise-induced thrombocytosis and subsequently increase circulating BDNF.

Small muscle mass exercise increases circulating catecholamines (Stratton et al., 1983), as well as muscle sympathetic nerve activity (Joyner et al., 1992) in an intensity-dependent manner. Frances et al. (2008) have provided preliminary evidence for splenic constriction and an associated small thrombocytosis response following 1 min of 40% maximal voluntary contraction (MVC) isometric forearm contraction. As such, it is plausible that performing small muscle mass exercise, such as, forearm handgrip exercise, could stimulate thrombocytosis and consequently achieve significant elevations in serum BDNF. Such an exercise protocol could stand as a viable means of transiently increasing a dose of BDNF without performing whole body exercise for individuals with mobility constraints or performed concurrently while in sedentary, occupational setting. However, no work to date has investigated this possibility.

Therefore, the purpose of this study was to determine the effect of two different forearm exercise modalities (maximal effort, short duration and submaximal effort, longer duration) on circulating BDNF and platelet levels. We hypothesized that maximal effort exercise (ME) would significantly elevate serum BDNF and platelet levels compared to rest and compared to submaximal effort exercise (SE), as ME would result in greater sympathetic activation, and therefore thrombocytosis, compared to SE.

## Methods

### Subjects

Eighteen healthy males (21.4 ± 2.1 years, height 180.5 ± 2.8 cm, weight 81.4 ± 4.7 kg, BMI 25.1 ± 1.0 kg/m^2^) were recruited for this study with no history of cardiovascular disease, hypertension, asthma, depression, smoking, or forearm-specific training. Participants were not taking prescription medication at the time of the study. This study was carried out in accordance with the recommendations of the Health Sciences Research Ethics Board at Queen's University, with written informed consent from all subjects. All subjects gave written informed consent in accordance with the Declaration of Helsinki. The protocol was approved by the Health Sciences Research Ethics Board at Queen's University.

### Experimental protocols

This study examined the response of serum BDNF to short-duration maximal and long-duration submaximal forearm handgrip exercise. Experimental protocols were performed on separate days and a 1 week washout period was enforced to prevent potential crossover effects from the previous session. Participants were instructed to refrain from exercise for 24 h, caffeine and alcohol 16 h, and food 8 h prior to experimental visits. All experimental sessions took place in the morning, between the hours of 8:00 and 10:00 a.m., to avoid variability in serum BDNF levels due to diurnal fluctuations (Katoh-Semba et al., 2007).

### Exercise protocols

Forearm handgrip exercise consisted of rhythmic isometric contractions of the forearm at a 2:2 s contraction: relaxation duty cycle. Handgrip exercise was performed using an electric handgrip dynamometer (AD Instruments, Colorado Springs, USA) with visual feedback of real-time force output displayed on a computer screen for participants.

The maximal effort (ME) protocol was 10 min in duration, during which participants performed MVCs for each contraction. Participants performed three MVC efforts separated by 1 min of rest prior to commencement of the ME protocol. Following completion of the MVC trial, a researcher set a visual target that participants were asked to attempt to reach with every contraction. A 10 min rest period was provided following completion of the MVC trial to ensure full recovery prior to commencement of the ME trial (Kellawan and Tschakovsky, 2014). The ME exercise protocol is characterized by an exponential decline in force production that eventually plateaus at what is termed critical impulse for such isometric exercise (Kellawan and Tschakovsky, 2014). Critical impulse is the force analog of critical power (i.e., is equivalent to critical power) which represents the maximal work rate supported by aerobic metabolism at which a metabolic steady state can be achieved (Kellawan and Tschakovsky, 2014). For our purposes, force output during the last min of exercise was analyzed and used for the determination of the submaximal exercise (SE) protocol exercise intensity. Participants received continual verbal encouragement from researchers to ensure maximal effort on every contraction.

For the SE protocol, participants performed 30 min of handgrip exercise at an intensity set at 15% below their estimated critical impulse. A visual target was displayed on a computer screen and participants were instructed to hit the target on every contraction. In both exercise protocols, venous blood draws were taken following 30 min of quiet rest (rest sample) and during the last 30 s of exercise (exercise sample). Exercise was well tolerated and all participants were able to complete the exercise sessions.

### Hemodynamic monitoring as a surrogate measure of autonomic activation

Hemodynamic variables, including arterial blood pressure, total peripheral resistance (TPR), cardiac output (CO), and heart rate (HR) responses to the experimental protocols were continually monitored on a beat-by-beat basis using finger photophlethysmography using the ModelFlow method (Finometer MIDI, Finapres Medical Systems, Amsterdam, Netherlands) on the non-exercising hand. These measures were used as a surrogate for sympathetic activity, as muscle sympathetic nerve activity parallels diastolic blood pressure (DBP) during handgrip exercise (Sander et al., 2010; Sayed et al., 2016).

### Catheterization and blood sampling/treatment

A vein of the exercising forearm was catheterized using a 20-gauge Teflon catheter (BD Insyte Autoguard, Oakville, Canada) upon entry into the lab. In all experimental protocols participants rested in a supine position for at least 30 min to avoid variability in plasma volume associated with postural changes (Kargotich et al., 1998). Blood for platelet determination was collected in EDTA K2 3 mL vacutainers and was analyzed by complete blood count by the CORE Lab at Kingston General Hospital (Kingston, Canada). Blood for serum BDNF determination was collected in serum collector tubes and left to clot at room temperature for 30 min and then at 4°C for an additional 30 min. Following the clotting period, samples were centrifuged at 2,000 × g for 15 min at 4°C, after which the supernatant was aliquoted and stored at −80°C.

### Biochemical analysis

Serum BDNF was analyzed using the BDNF Emax Immunoassay System (Promega, Madison, WI, USA). The kit detection range was 7.8 pg/mL —500 pg/mL and the intra-assay coefficient of variation was 2.9%. The coating buffer was set to a pH of 9.7 and pipetted in each well of an Immulon 4 HBX Extra High Binding 96-well plate (Thermo Scientific, Rochester, NY, USA) and incubated overnight at 4°C. Serum samples were diluted by a factor of 1/128 with the prepared 1 × block and sample buffer and run in duplicate. All incubation times and washing steps were followed in accordance with the manufacturer's instructions. Following the addition of tetramethylbenzidine substrate (color development step), samples were incubated at room temperature for 10 min, upon which 1 mmol hydrochloric acid was added to stop color development. Following this, the plate was immediately read at 450 nm using an automated plate reader (Synergy 2, BioTek Instruments, Winooski, VT, USA). A standard curve was generated using a linear regression curve-fit approach with the plate reader software and all standard curves *r*^2^ values were between 0.99 and 1.00. All samples were subsequently multiplied by a dilution factor of 128. BDNF samples were analyzed within 4 months of collection.

### Handgrip data acquisition and analysis

Handgrip exercise was performed using an electronic handgrip dynamometer (ADInstruments, Colorado Springs, USA) and force output was recorded via data acquisition software on a lab computer (Powerlab, ADInstruments, Colorado Springs, USA). MVC peak force was determined as the highest point of a handgrip contraction impulse performed during MVC testing at the beginning of the ME session. All contractions performed during the ME and SE conditions were quantified using a time-tension integral of the force tracing and an average impulse force output was obtained by dividing total impulse force by the number of contractions performed, and further dividing by two given each contraction was 2 s in duration.

### Statistical analysis and sample size determination

A two-way repeated measured analysis of variance was used to determine differences in BDNF, platelet, and hemodynamic variables at rest and during exercise in ME and SE testing sessions. Given that changes in plasma volume that occur with exercise can directly impact the concentration of measured blood constituents, we calculated a 5 and 3% decrease in plasma for ME and SE, respectively, based on the method of Dill and Costill (Dill and Costill, 1974). Blood samples from one participant were misplaced by the hematological lab that ran our platelet analysis, as such we report platelets values for 17 participants in response to ME and SE. A Bonferroni correction for multiple comparisons was performed where appropriate. A two-tailed paired *t*-test was used to compare the total amount of work performed between ME and SE. Further, a two-tailed paired *t*-test was also used to compare average handgrip impulse force between conditions. Pearson product-moment correlation coefficients were calculated to determine relationships between DBP (surrogate for sympathetic activation), platelets, and BDNF. All analysis was performed using SigmaPlot Statistical Analysis and Scientific Graphing Software version 12.0. All data are expressed as mean ± standard deviation (SD) with statistical significance set at *p* ≤ 0.05. Sample size was calculated using a two-tailed paired *t*-test with α set at 0.05 and desired power of 0.95. Using the baseline serum BDNF values reported by Ferris et al. (2007; 18,168 ± 1,193 pg/mL) with a desired minimum increase of 10%, we determined the required sample size for this study was *n* = 18.

## Results

### BDNF responses to forearm handgrip exercise

**Figure 1:**
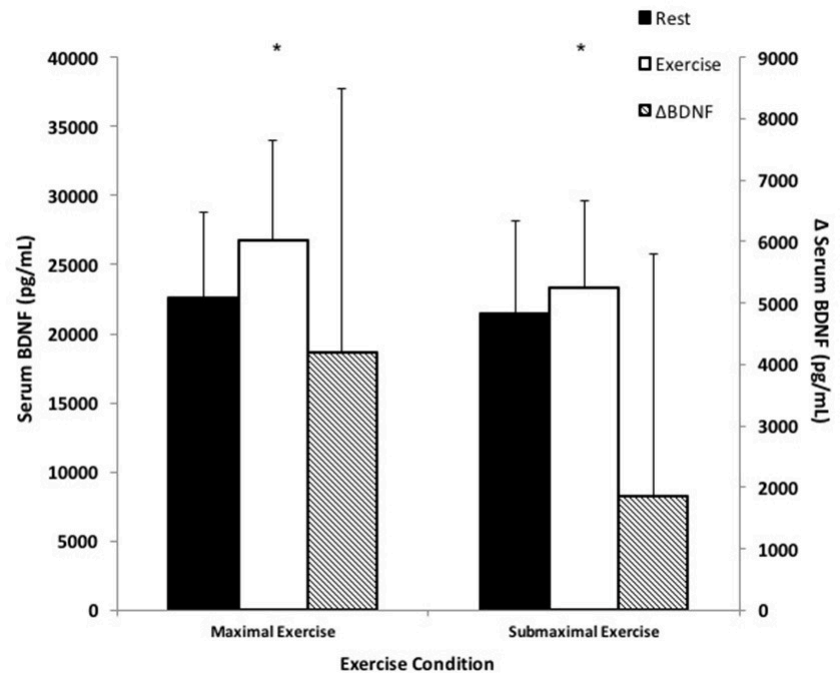
BDNF responses to small muscle mass exercise. Total serum BDNF at rest (black bars) and total serum BDNF during exercise (white bars) correspond with the left y-axis. ΔBDNF represents the difference between exercise and rest BDNF (hatched bars) and corresponds with the right y-axis. ^*^Significantly different compared to rest, *p* < 0.05.

There was a main effect of time as serum BDNF significantly increased in response to exercise compared to rest in both exercise conditions (Figure 1). Specifically, BDNF increased by 21.2 ± 24.5% in response to ME; and 11.2 ± 21.2% in response to SE; *F*_(1, 16)_ = 14.59, *p* < 0.05. There was a statistically non-significant trend toward a time × condition interaction with ME being greater than SE post-exercise BDNF; *F*_(1, 16)_ = 4.06, *p* = 0.06.

### Platelet responses to forearm handgrip exercise

**Figure 2:**
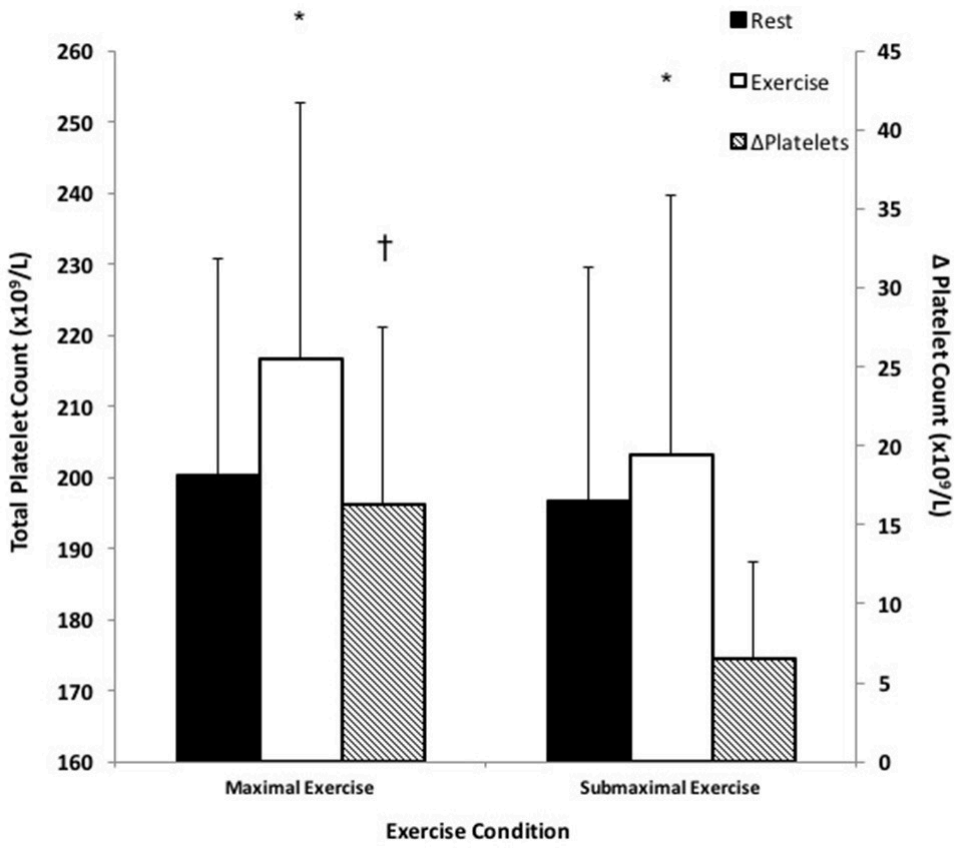
Platelet responses to small muscle mass exercise. Total platelets at rest (black bars) and total platelets during exercise (white bars) correspond with the left y-axis. ΔPlatelets represents the difference between exercise and rest platelets (hatched bars) and corresponds with the right y-axis. ^*^Significantly different compared to rest, *p* < 0.05. ^†^Significantly different compared to SE, *p* < 0.05.

There was a significant time × condition interaction as platelets significantly increased in response to both exercise conditions compared to pre-exercise measures, *F*_(1, 15)_ = 17.75, *p* < 0.05 (Figure 2). Specifically, ME stimulated an 8.0 ± 5.2% increase in platelets, which was a significantly greater response compared to SE, which stimulated a 3.1 ± 3.5% increase in platelets; *F*_(1, 15)_ = 34.73, *p* < 0.05.

### BDNF per platelet analysis

**Figure 3:**
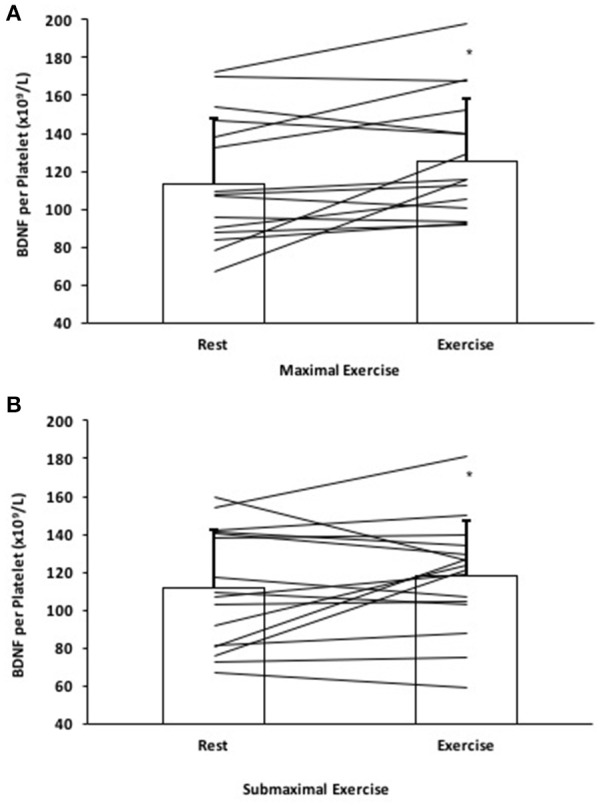
BDNF per platelet response to **(A)** Maximal effort exercise and **(B)** Submaximal effort exercise. Individual responses denoted by straight lines and group means represented by bars. ^*^Significantly different compared to rest, *p* < 0.05.

The calculated amount of BDNF per platelet significantly increased by 13.4 ± 23.6% following ME and a 8.7 ± 23.2% increase in BDNF per platelet following SE; *F*_(1, 15)_ = 4.60, *p* < 0.05 (Figure 3).

### Hemodynamic responses to exercise

Table 1 displays the hemodynamic responses to ME and SE protocols. There was an effect of time in both exercise conditions for all variables except for TPR, *p* < 0.05. There was also an effect of condition, as ME was significantly greater than SE across all variables except for CO and TPR, *p* < 0.05.

**Table 1:** Hemodynamic responses to exercise.

	**ME**		**SE**	
	**Rest**	**Exercise**	**Rest**	**Exercise**
SBP (mmHg)	125 (12)	153 (12)^*^^†^	121 (7)	140 (8)^*^
DBP (mmHg)	72 (7)	97 (10)^*^^†^	66 (10)	82 (6)^*^
MAP (mmHg)	90 (9)	121 (10)^*^^†^	84 (9)	105 (8)^*^
CO (L/min)	6.0 (1.2)	8.6 (1.5)^*^	6.0 (1.2)	7.5 (1.2)^*^
TPR (mmHg/mL/min)	0.95 (0.20)	0.91 (0.19)	0.88 (0.20)	0.86 (0.14)
HR (bpm)	59.5 (10.5)	95.3 (15.9)^*^^†^	55.9 (7.4)	70.2 (7.4)^*^

*SBP, systolic blood pressure; DBP, diastolic blood pressure; MAP, mean arterial pressure; CO, cardiac output; TPR, total peripheral resistance; HR, heart rate; Data are mean (SD)*.

* *Significantly different than Rest of the same condition*.

† *Significantly greater than SE*.

### Correlations between DBP, platelets, and BDNF

**Figure 4:**
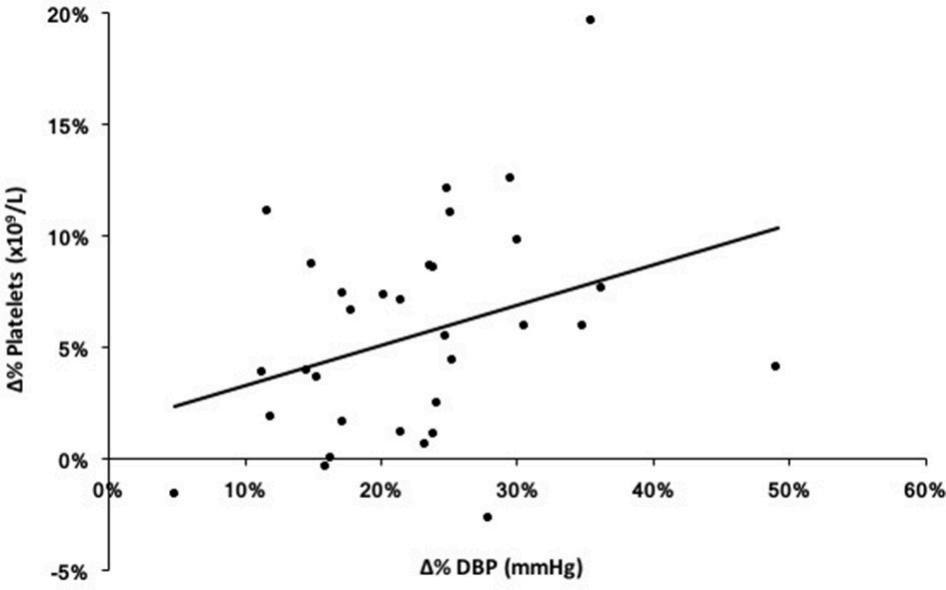
Exploratory correlation between percent change (Δ%) in diastolic blood pressure (DBP) and platelets in response to both ME and SE (pooled exercise condition). *r* = 0.363, *p* < 0.05.

Pearson product-moment correlation coefficients were calculated for DBP, platelets, and BDNF for each exercise condition and pooled data from both exercise protocols (Figure 4). There was a significant association between DBP and platelets in the pooled exercise conditions, *r* = 0.363, *p* < 0.05. There were no associations found between BDNF and platelets or DBP in response to exercise.

### Handgrip exercise performance and total work performed

Given the differences in both intensity and duration of the respective exercise protocols, the total volume of work performed was analyzed. The SE protocol resulted in significantly greater total work performed (154,508.4 ± 21,673.4 N) compared to ME (75,282.2 ± 15,342.9 N), *p* < 0.05. The average critical power impulse for the group was 192.5 ± 35.5 N•s, from which an average target of 163.6 ± 30.2 N•s corresponding to 15% below critical power was set as the SE intensity. The average handgrip impulse force during ME was 225.2 ± 72.0 N•s, which was significantly greater than SE (164.4 ± 25.1 N•s), *p* < 0.05.

## Discussion

The guiding rationale for this study was that the robust increase in serum BDNF following aerobic exercise of sufficient intensity is in part due to the addition of splenic platelets, via sympathetically mediated splenic constriction. As such, we hypothesized that forearm handgrip exercise could increase serum BDNF in an intensity-dependent manner due to increased sympathetic activation evoked by exercise (Stratton et al., 1983; Joyner et al., 1992). The major findings from this study were (a) serum BDNF significantly increased in response to both exercise protocols, (b) platelets significantly increased in an intensity-dependent manner, with ME evoking a larger response compared to SE, and (c) the amount of BDNF per platelet significantly increased in both exercise conditions. These findings support our hypothesis and to our knowledge are the first to demonstrate a significant serum BDNF response can be achieved with as little exercising muscle mass as a single forearm.

### BDNF response to exercise: intensity and muscle mass considerations

Ten minutes of ME and thirty minutes of SE elicited 21.2 and 11.2% increases in serum BDNF, respectively. Our small muscle mass exercise protocols were designed based on the rationale that elevated sympathetic nervous activity was a required effect of exercise in order to induce thrombocytosis and thereby achieve serum BDNF elevation. Prior studies that did use a small muscle mass resistance exercise model did not consider the nature of exercise in this context (Correia et al., 2010; Rojas Vega et al., 2010). Potential explanations for the differences between these and our findings reveal themselves when we consider exercise protocols in the context of activating specific mechanisms of BDNF addition to the blood. Specifically, insufficient intensity and/or duration could explain the lack of findings in these studies (Correia et al., 2010; Rojas Vega et al., 2010) compared to the robust serum BDNF increase in our forearm handgrip exercise model. This is because of the need for sufficient magnitude and duration of peripheral stimuli like shear stress or deoxygenation for endothelial or brain BDNF production and release (Rasmussen et al., 2009; Prigent-Tessier et al., 2013), and sympathetic splenic vasoconstriction for thrombocytosis. The former was recently proposed by Marston et al. (2017) to explain the finding that only a strength training bout for the purpose of hypertrophy provided enough stimulus to achieve elevations in serum BDNF. In support of the latter, we did obtain blood samples at 3 min in a small subset (*n* = 5) of subjects and found at that time point that there was a limited although non-statistically significant (*P* = 0.053) increase in platelets compared to baseline for ME (188.3 ± 23.3 vs. 196.3 ± 23.1 × 10^9^/L) and no evidence of an increase in SE (190.3 ± 25.8 vs. 189.3 ± 21.7 × 10^9^/L). By comparison, the full exercise protocols in our study required participants to complete 300 contractions for ME for a duration of 10 min and 900 contractions over a period of 30 min for SE, at which time we saw robust serum BDNF increases. Furthermore, we (Walsh et al., 2016) and others (Yarrow et al., 2010; Marston et al., 2017) have reported significant increases in serum BDNF following resistance exercise protocols consisting of multiple exercises and lasting longer than 3 min (i.e., 45 min including rest). We would emphasize the need for activation of specific BDNF elevating mechanisms to be a prime consideration when assessing the potential efficacy of an exercise modality.

The body of evidence with regard to aerobic exercise as summarized by Knaepen et al. (2010) suggests that circulating BDNF increases in an intensity-dependent fashion, with the largest BDNF responses occurring at higher aerobic exercise intensities in healthy populations. The magnitude of the BDNF response observed in our study using a single forearm handgrip exercise was expectedly lower than whole-body high intensity exercise protocols, such as, Cho et al. (2012) who observed a 39% increase in serum BDNF at the group level following a VO_2_max treadmill test, and Gilder et al. (2014) and Ferris et al. (2007) who noted a 32 and 30% increase in serum BDNF in response to a maximal exercise test on a cycle ergometer, respectively. We have demonstrated that 10 min of ME forearm exercise achieved an ~21% increase in serum BDNF, which is 2/3 of the response observed by Gilder et al. (2014) and Ferris et al. (2007). Based on previous work, and without considering splenic constriction as a key mechanism for achieving elevated serum BDNF, this would seem a remarkable effect given that forearm muscle mass is a mere fraction of that used during maximal cycling or running exercise. Thus, we believe our work is the first to demonstrate that stimulation of thrombocytosis independent of muscle mass activation is an important consideration for an exercise modality's efficacy in elevating circulating BDNF.

Interestingly, the work by Ferris et al. (2007) alludes to the possibility of an intensity threshold for achieving circulating BDNF elevation. They found that cycling exercise at 75% VO_2_max evoked a 13% increase in serum BDNF, whereas cycling at 55% VO_2_max did not have an effect on BDNF following exercise. This finding is supported by those of Rojas Vega et al. (2006) and Hötting et al. (2016) and recently emphasized by Marston et al. (2017) for resistance exercise. In the present study, although not statistically significant between ME and SE (*P* = 0.06) the serum BDNF response, in conjunction with the significantly greater platelet response in ME, encourage consideration of a relationship between exercise intensity and serum BDNF elevation that, when viewed in conjunction with the findings of Ferris et al. (2007) may be somewhat independent of muscle mass. The potential for this independence was in fact the basis for our hypothesis that forearm handgrip exercise could achieve significant elevations in circulating BDNF, given that the magnitude of local skeletal muscle metabolic stress appears to be a critical driver of sympathetic nerve activity independent of muscle mass (Saito and Mano, 1991).

### Platelet source of BDNF in response to exercise

To reiterate, the rationale of the current study was that sympathetic activity evoked by handgrip exercise would cause splenic constriction, thereby resulting in the ejection of stored platelets. Given that ~99% of circulating BDNF is platelet-bound, this thrombocytosis could account for a large part of the robust serum BDNF increase following acute exercise, even if only 30–40% of this BDNF is releasable (Fujimura et al., 2002; Tamura et al., 2011). In support of this relationship, we found a significant correlation between platelet and DBP responses to exercise, given that DBP and muscle sympathetic nerve activity respond in parallel to handgrip exercise (Sander et al., 2010; Sayed et al., 2016). There are two interesting parallels between BDNF and platelet responses to exercise that suggest a primary role for thrombocytosis in BDNF elevation. The first is that both are exercise intensity-dependent phenomena (Gimenez et al., 1986; Knaepen et al., 2010) and the second commonality is that both share similar post-exercise characteristics (Brunelli et al., 2012; Gilder et al., 2014). While we did not include post-exercise measures, previous works have independently shown a return to baseline within 30 min of exercise cessation for both platelets (Brunelli et al., 2012) and serum BDNF (Rojas Vega et al., 2006; Matthews et al., 2009; Gilder et al., 2014), a response that is likely the result of splenic reuptake of platelets due to sympathetic withdrawal (Chamberlain et al., 1990; Stewart et al., 2003).

### Tissue sources of BDNF in response to exercise

Serum BDNF and platelet count are strongly linked (Fujimura et al., 2002; Matthews et al., 2009; Nettiksimmons et al., 2014); however, correlational analysis revealed no relationship between changes in platelets and BDNF for either of our exercise conditions, despite both moving in the same direction. This can be explained by the possibility of a tissue source contributing *de novo* BDNF to the circulation, as we observed an increase in BDNF per platelet in ME and SE exercise conditions. An increase in BDNF per platelet cannot be the function of endogenous platelet BDNF production, as platelets do not synthesize the neurotrophin (Fujimura et al., 2002). Nor is it likely that splenic platelets have a greater BDNF content than circulating platelets, given that they exist in dynamic equilibrium undergoing constant exchange (Wadenvik and Kutti, 1988; Chamberlain et al., 1990).

The seminal study by Rasmussen et al. (2009) implicating the brain as the primary source of circulating BDNF dominates the current framework by which exercise-induced elevation of blood borne BDNF via tissue source is understood. However, their conclusions that 70–80% of circulating BDNF is of cerebral origin may overestimate the brain's contribution to circulating BDNF as these authors only measured plasma and it was specifically venous effluent immediately downstream of the brain. The major issue with tissue effluent plasma as the lone measure of BDNF, and its interpretation with regard to tissue production and release is that BDNF can move between platelets and plasma in response to local vascular bed elevations in shear-stress (Fujimura et al., 2002). As this is elevated in the cerebral circulation during exercise (Seifert and Secher, 2011), there exists the conditions for increased platelet release of BDNF in the cerebral microcirculation. This suggests that caution should be used in interpreting tissue venous effluent plasma-only measures of BDNF, as both tissue and platelet release contributions are possible.

Other tissues that produce and release BDNF in response to exercise-like stimuli have also been identified, including the vascular endothelial cells (VEC) (Prigent-Tessier et al., 2013) and peripheral blood mononuclear cells (PBMC) (Brunelli et al., 2012). VEC have recently been shown to rapidly secrete BDNF in proportion to the magnitude of shear stress stimuli (Prigent-Tessier et al., 2013) and reductions in PO_2_ (Helan et al., 2014), both of which occur in considerable measure in exercising skeletal muscle. In fact, Prigent-Tessier et al. (2013) report that levels of pro and mature BDNF in cardiac and aortic endothelium were in the same range as those measured in the hippocampus (Quirié et al., 2012), highlighting that the cardiovascular system is a likely contributor to exercise-induced elevation in circulating BDNF. To what extent this source was activated by forearm handgrip exercise remains unclear, given the small exercising muscle mass. In summary, both splenic-specific elevated platelet BDNF content and release and tissue *de novo* BDNF production remain potential contributors to the observed increase in BDNF per platelet in our study.

### Perspectives

This is the first study to demonstrate that it only takes a single forearm exercising muscle mass to stimulate increases in serum BDNF similar to those levels seen in high to moderate intensity traditional aerobic paradigms. In fact, the 10 min of ME forearm exercise resulted in 2/3 of the serum BDNF response evoked by a whole body graded exercise test to exhaustion as seen by Ferris et al. (2007). The important implications of these findings are two-fold. First, these results provide insight into potential mechanisms by which exercise may increase circulating BDNF. Whole-body aerobic exercise creates a substantial multi-faceted physiological perturbation (i.e., increased shear stress, tissue deoxygenation, increased neuronal firing, hyperthermia, and increase sympathetic outflow), that could evoke a coordinated response from multiple systems that are potential sources of BDNF. Evidence from cell studies suggests the magnitude of tissue BDNF release is proportional to the physiological perturbations such as, shear stress (Prigent-Tessier et al., 2013), tissue deoxygenation (Helan et al., 2014), and chemical signaling on PBMCs (Brunelli et al., 2012); conditions which would be present in exercising muscle. In this context, the exercising forearm muscle mass would represent but a fraction of such an endothelial source compared to cycling or running. Therefore, it would seem at first glance that such small muscle mass exercise could not significantly elevate serum BDNF. However, based on our findings it would appear that constriction of the spleen and the subsequent thrombocytosis is an important mechanism for elevating BDNF during exercise and forearm exercise provides ample stimulus for this. The major differentiator between forearm and whole-body exercise may therefore be the contribution of cellular sources to total circulating BDNF. We found a 13.5% increase in BDNF per platelet following maximal effort forearm exercise compared to a calculated 59.7% increase following a treadmill VO_2_max test (Cho et al., 2012). In the context of the current body of literature, our findings are important because they identify the potential for exercise modality-specific mechanisms of BDNF elevation, and direct follow up research investigation into issues of platelet-bound BNDF bioavailability at tissue exchange sites and its importance, and the potential of sympathetic activation interventions to contribute.

The second important aspect of this study pertains to developing strategies for increasing circulating BDNF in populations that stand to benefit most from this transient response. The relationship between circulating BDNF and brain function has been demonstrated in populations with depression and in aging populations (Ziegenhorn et al., 2007; Brunoni et al., 2008). In both cases, exercise has been touted as an effective strategy for restoring and improving function and BDNF appears to be a central mediator of this relationship. It follows that finding easy yet effective ways of transiently increasing BDNF may have important functional implications for such populations and warrants further investigation.

We also believe it important to recognize that, while serum BDNF elevation is transient following exercise, this does not mean that significant impact on neuroplasticity cannot be achieved. We have previously put forward the notion that transient exercise-induced elevations in circulating neurotrophic factors could be taken advantage of by performing cognitive training immediately following exercise (Walsh et al., 2016). The concept is that the increased cerebral perfusion due to neurovascular coupling with brain activation would benefit from having increased blood borne BDNF as a means of increasing the BDNF “dose” to brain areas important in cognitive function. Again it is important to stress that platelet-bound BDNF should be considered bioavailable at the site of tissue uptake as platelets release BDNF under conditions of increased shear stress (Fujimura et al., 2002), which would be the case in areas of increase cerebral perfusion. As well, elevated epinephrine accompanying adrenergic activating exercise intensities increases such a release response. To draw an analogy; greater hemoglobin content accompanied by decreased affinity of Hb for oxygen, ensures a higher tissue microvascular availability of oxygen for concentration dependent diffusion. Likewise greater platelet content accompanied by increased reactivity to tissue exchange site stimuli may serve to enhance the microvascular plasma concentration of BDNF and thereby the transporter dependent uptake by neural tissue (Pan et al., 1998). It may be the case that such transient but periodic increases in BDNF uptake as a result of regular exercise bouts are an important contributor to neuroplasticity.

### Limitations

We do not report plasma measures of BDNF in this study. We had intended to do so as samples were collected and processed for the determination of plasma BDNF; however, the optical density signal in these plasma samples was below that of the lowest standard, rendering these samples unmeasurable. Unfortunately, this is not an uncommon occurrence, as Brunelli et al. (2012) and Gilder et al. (2014) both report difficulties in the measurement of plasma samples. However, given that the overwhelming majority of blood borne BDNF is platelet bound and the established interchange of BDNF between plasma and platelets, the measurement of serum and BDNF/platelet provide a meaningful characterization of the BDNF system that captures both splenic and tissue sources of BDNF. It should be noted that in contrast to plasma samples the optical density signal of serum samples was in the optimal kit detection range and there was therefore no difficulty in measuring serum BDNF.

We report that the amount of BDNF per platelet increased following both exercise protocols; however, this measure is calculated from serum BDNF divided by platelet count rather than a direct measurement from lysed platelets. While this measure may limit direct quantitative comparison between exercise conditions, it does allow for the identification of a direction of change in platelet bound BDNF.

Our study exercise modalities were not originally intended to control for exercise volume across exercise intensities and therefore it must be acknowledged that greater platelet and BDNF elevation in ME than SE may also be explained by the response dwindling over time with continued steady state forearm exercise in SE rather than because of intensity differences between ME and SE. This seems unlikely given that exercise intensity has a greater impact on the magnitude of thrombocytosis than exercise duration (Gimenez et al., 1986). It is also possible that with longer duration, increased tissue uptake might begin to reduce circulating BDNF (Marston et al., 2017). Regardless, our findings are the first to support the efficacy of single forearm exercise to elevate circulating BDNF to levels similar to those observed in moderate to high intensity whole body aerobic exercise (Knaepen et al., 2010) and encourage further investigation into muscle mass independent exercise intensity effects on mechanisms of circulating BDNF elevation.

Finally, this study only examined males, which limits the generalizability of our findings given that males appear to have stronger BDNF responses to exercise than females (Szuhany et al., 2015). As such, follow-up work should be undertaken to examine the response in BDNF response to handgrip exercise in both males and females.

## Conclusions

In conclusion, this is the first study to demonstrate that exercise with as little muscle mass as contained in a forearm can significantly increase circulating BDNF. Furthermore, this appears to be sensitive to exercise intensity (*p* = 0.06 for differences between ME and SE). This substantial BDNF response is associated with an intensity-dependent increase in circulating platelets indicative of thrombocytosis that we interpret as being a major source of exercise-induced BDNF in a forearm handgrip model. As well, the observation that calculated BDNF per platelet significantly increased could implicate the contribution of a cellular source of BDNF in response to handgrip exercise and/or higher BDNF content of splenic-derived platelets. These findings support the potential for forearm handgrip exercise as an intervention capable of achieving significant increases in serum BDNF. They also provide the rationale for pursuing new lines of research into the role of sympathetic activation in BDNF bioavailability, and introduce an emphasis on thrombocytosis as an important response for an exercise modality that is accessible to most populations, requires minimal equipment (i.e., stress ball), and requires considerably less effort than conventional exercise.

## Author contributions

JW and BG were responsible for performing biochemical assay optimization. JW was responsible for the collection, processing, and analysis of all experimental data. BG oversaw biochemical analysis. JW and RB were responsible for running experimental sessions. JW, BG, and MT were for responsible for the conception, design, and interpretation of the experiments. JW and MT wrote the manuscript, and JW, BG, RB, and MT revised the manuscript. All authors approved the final version of the manuscript.

### Conflict of interest statement

The authors declare that the research was conducted in the absence of any commercial or financial relationships that could be construed as a potential conflict of interest.

## References

[B1] Ahmadizad S. El-Sayed M. S. Maclaren D. P. (2006). Responses of platelet activation and function to a single bout of resistance exercise and recovery. Clin. Hemorheol. Microcirc. 35, 159–168. 16899922

[B2] Angelucci F. Gelfo F. De Bartolo P. Caltagirone C. Petrosini L. (2011). BDNF concentrations are decreased in serum and parietal cortex in immunotoxin 192 IgG-Saporin rat model of cholinergic degeneration. Neurochem. Int. 59, 1–4. 10.1016/j.neuint.2011.04.010 21672569

[B3] Bakovic D. Pivac N. Eterovic D. Breskovic T. Zubin P. Obad A.. (2013). The effects of low-dose epinephrine infusion on spleen size, central and hepatic circulation and circulating platelets. Clin. Physiol. Funct. Imaging 33, 30–37. 10.1111/j.1475-097X.2012.01156.x 23216763

[B4] Brunelli A. Dimauro I. Sgrò P. Emerenziani G. P. Magi F. Baldari C.. (2012). Acute exercise modulates BDNF and pro-BDNF protein content in immune cells. Med. Sci. Sports Exerc. 44, 1871–1880. 10.1249/MSS.0b013e31825ab69b 22543740

[B5] Brunoni A. R. Lopes M. Fregni F. (2008). A systematic review and meta-analysis of clinical studies on major depression and BDNF levels: implications for the role of neuroplasticity in depression. Int. J. Neuropsychopharmacol. 11, 1169–1180. 10.1017/S1461145708009309 18752720

[B6] Chamberlain K. G. Tong M. Penington D. G. (1990). Properties of the exchangeable splenic platelets released into the circulation during exercise-induced thrombocytosis. Am. J. Hematol. 34, 161–168. 10.1002/ajh.2830340302 2363410

[B7] Cho H. C. Kim J. Kim S. Son Y. H. Lee N. Jung S. H. (2012). The concentrations of serum, plasma and platelet BDNF are all increased by treadmill VO_2_max performance in healthy college men. Neurosci. Lett. 519, 78–83. 10.1016/j.neulet.2012.05.025 22617010

[B8] Correia P. R. Pansani A. Machado F. Andrade M. Silva A. C. Scorza F. A.. (2010). Acute strength exercise and the involvement of small or large muscle mass on plasma brain-derived neurotrophic factor levels. Clinics 65, 1123–1126. 10.1590/S1807-59322010001100012 21243284 PMC2999707

[B9] Cotman C. W. Berchtold N. C. Christie L. A. (2007). Exercise builds brain health: key roles of growth factor cascades and inflammation. Trends Neurosci. 30, 464–472. 10.1016/j.tins.2007.06.011 17765329

[B10] Dill D. B. Costill D. (1974). Calculation of percentage changes in volumes of blood, plasma, and red cells in dehydration. J. Appl. Physiol. 37, 247–248. 4850854 10.1152/jappl.1974.37.2.247

[B11] Erickson K. I. Prakash R. S. Voss M. W. Chaddock L. Heo S. McLaren M.. (2010). Brain-derived neurotrophic factor is associated with age-related decline in hippocampal volume. J. Neurosci. 30, 5368–5375. 10.1523/JNEUROSCI.6251-09.2010 20392958 PMC3069644

[B12] Ferris L. T. Williams J. S. Shen C. L. (2007). The effect of acute exercise on serum brain-derived neurotrophic factor levels and cognitive function. Med. Sci. Sports Exerc. 39, 728–734. 10.1249/mss.0b013e31802f04c7 17414812

[B13] Frances M. F. Dujic Z. Shoemaker J. K. (2008). Splenic constriction during isometric handgrip exercise in humans. Appl. Physiol. Nutr. Metab. 33, 990–996. 10.1139/H08-087 18923575

[B14] Fujimura H. Altar C. A. Chen R. Nakamura T. Nakahashi T. Kambayashi J. I.. (2002). Brain-derived neurotrophic factor is stored in human platelets and released by agonist stimulation. Thromb. Haemost. 87, 728–734. 12008958

[B15] Gilder M. Ramsbottom R. Currie J. Sheridan B. Nevill A. M. (2014). Effect of fat free mass on serum and plasma BDNF concentrations during exercise and recovery in healthy young men. Neurosci. Lett. 560, 137–141. 10.1016/j.neulet.2013.12.034 24368215

[B16] Gimenez M. Mohan-Kumar T. Humbert J. C. De Talance N. Buisine J. (1986). Leukocyte, lymphocyte and platelet response to dynamic exercise - Duration or intensity effect? Eur. J. Appl. Physiol. Occup. Physiol. 55, 465–470. 10.1007/BF00421638 3769902

[B17] Helan M. Aravamudan B. Hartman W. R. Thompson M. A. Johnson B. D. Pabelick C. M.. (2014). BDNF secretion by human pulmonary artery endothelial cells in response to hypoxia. J. Mol. Cell. Cardiol. 68, 89–97. 10.1016/j.yjmcc.2014.01.006 24462831 PMC3977651

[B18] Hötting K. Schickert N. Kaiser J. Röder B. Schmidt-Kassow M. (2016). The effects of acute physical exercise on memory, peripheral BDNF, and cortisol in young adults. Neural Plast. 2016:6860573. 10.1155/2016/6860573 27437149 PMC4942640

[B19] Hulmi J. J. Myllymäki T. Tenhumäki M. Mutanen N. Puurtinen R. Paulsen G.. (2010). Effects of resistance exercise and protein ingestion on blood leukocytes and platelets in young and older men. Eur. J. Appl. Physiol. 109, 343–353. 10.1007/s00421-010-1360-7 20101405

[B20] Jørgensen L. G. Perko G. Secher N. H. (1992). Regional cerebral artery mean flow velocity and blood flow during dynamic exercise in humans. J. Appl. Physiol. 73, 1825–1830. 1474058 10.1152/jappl.1992.73.5.1825

[B21] Joyner M. J. Nauss L. A. Warner M. A. Warner D. O. (1992). Sympathetic modulation of blood flow and O_2_ uptake in rhythmically contracting human forearm muscles. Am. J. Physiol. 263, H1078–H1083. 1415755 10.1152/ajpheart.1992.263.4.H1078

[B22] Karege F. Bondolfi G. Gervasoni N. Schwald M. Aubry J. M. Bertschy G. (2005). Low Brain-Derived Neurotrophic Factor (BDNF) levels in serum of depressed patients probably results from lowered platelet BDNF release unrelated to platelet reactivity. Biol. Psychiatry 57, 1068–1072. 10.1016/j.biopsych.2005.01.008 15860348

[B23] Kargotich S. Goodman C. Keast D. Morton A. R. (1998). The influence of exercise-induced plasma volume changes on the interpretation of biochemical parameters used for monitoring exercise, training and sport. Sports Med. 26, 101–117. 10.2165/00007256-199826020-00004 9777683

[B24] Katoh-Semba R. Wakako R. Komori T. Shigemi H. Miyazaki N. Ito H.. (2007). Age-related changes in BDNF protein levels in human serum: differences between autism cases and normal controls. Int. J. Dev. Neurosci. 25, 367–372. 10.1016/j.ijdevneu.2007.07.002 17804189

[B25] Kellawan J. M. Tschakovsky M. E. (2014). The single-bout forearm critical force test: a new method to establish forearm aerobic metabolic exercise intensity and capacity. PLoS ONE 9:e93481. 10.1371/journal.pone.0093481 24699366 PMC3974771

[B26] Knaepen K. Goekint M. Heyman E. M. Meeusen R. (2010). Neuroplasticity - exercise-induced response of peripheral brain-derived neurotrophic factor: a systematic review of experimental studies in human subjects. Sports Med. 40, 765–801. 10.2165/11534530-000000000-00000 20726622

[B27] Lommatzsch M. Zingler D. Schuhbaeck K. Schloetcke K. Zingler C. Schuff-Werner P.. (2005). The impact of age, weight and gender on BDNF levels in human platelets and plasma. Neurobiol. Aging 26, 115–123. 10.1016/j.neurobiolaging.2004.03.002 15585351

[B28] Marston K. J. Newton M. J. Brown B. M. Rainey-Smith S. R. Bird S. Martins R. N.. (2017). Intense resistance exercise increases peripheral brain-derived neurotrophic factor. J. Sci. Med. Sport 20, 899–903. 10.1016/j.jsams.2017.03.015 28511848

[B29] Matthews V. B. Aström M. B. Chan M. H. Bruce C. R. Krabbe K. S. Prelovsek O.. (2009). Brain-derived neurotrophic factor is produced by skeletal muscle cells in response to contraction and enhances fat oxidation via activation of AMP-activated protein kinase. Diabetologia 52, 1409–1418. 10.1007/s00125-009-1364-1 19387610

[B30] Nettiksimmons J. Simonsick E. M. Harris T. Satterfield S. Rosano C. Yaffe K. (2014). The associations between serum brain-derived neurotrophic factor, potential confounders, and cognitive decline: a longitudinal study. PLoS ONE 9:e91339. 10.1371/journal.pone.0091339 24670553 PMC3966768

[B31] Pan W. Banks W. A. Fasold M. B. Bluth J. Kastin A. J. (1998). Transport of brain-derived neurotrophic factor across the blood – brain barrier. Neuropharmacology 37, 1553–1561. 10.1016/S0028-3908(98)00141-5 9886678

[B32] Phillips C. Baktir M. A. Srivatsan M. Salehi A. (2014). Neuroprotective effects of physical activity on the brain: a closer look at trophic factor signaling. Front. Cell. Neurosci. 8:170. 10.3389/fncel.2014.00170 24999318 PMC4064707

[B33] Prigent-Tessier A. Quirié A. Maguin-Gaté K. Szostak J. Mossiat C. Nappey M.. (2013). Physical training and hypertension have opposite effects on endothelial brain-derived neurotrophic factor expression. Cardiovasc. Res. 100, 374–382. 10.1093/cvr/cvt219 24092446

[B34] Quirié A. Hervieu M. Garnier P. Demougeot C. Mossiat C. Bertrand N. (2012). Comparative effect of treadmill exercise on mature bdnf production in control vs. stroke rats. PLoS ONE 7:e44218 10.1371/journal.pone.0044218 22962604 PMC3433479

[B35] Radka S. F. Holst P. A. Fritsche M. Altar C. A. (1996). Presence of brain-derived neurotrophic factor in brain and human and rat but not mouse serum detected by a sensitive and specific immunoassay. Brain Res. 709, 122–130. 10.1016/0006-8993(95)01321-0 8869564

[B36] Rasmussen P. Brassard P. Adser H. Pedersen M. V. Leick L. Hart E.. (2009). Evidence for a release of brain-derived neurotrophic factor from the brain during exercise. Exp. Physiol. 94, 1062–1069. 10.1113/expphysiol.2009.048512 19666694

[B37] Rojas Vega S. Knicker A. Hollmann W. Bloch W. Strüder H. K. (2010). Effect of resistance exercise on serum levels of growth factors in humans. Horm. Metab. Res. 42, 982–986. 10.1055/s-0030-1267950 21053157

[B38] Rojas Vega S. Strüder H. K. Vera Wahrmann B. Schmidt A. Bloch W. Hollmann W.. (2006). Acute BDNF and cortisol response to low intensity exercise and following ramp incremental exercise to exhaustion in humans. Brain Res. 1121, 59–65. 10.1016/j.brainres.2006.08.105 17010953

[B39] Rosenfeld R. D. Zeni L. Haniu M. Talvenheimo J. Radka S. F. Bennett L.. (1995). Purification and identification of brain-derived neurotrophic factor from human serum. Protein Expr. Purif. 6, 465–471. 10.1006/prep.1995.1062 8527932

[B40] Saito M. Mano T. (1991). Exercise mode affects muscle sympathetic nerve responsiveness. Jpn. J. Physiol. 41, 143–151. 10.2170/jjphysiol.41.143 1857017

[B41] Sander M. Macefield V. G. Henderson L. A. (2010). Cortical and brain stem changes in neural activity during static handgrip and postexercise ischemia in humans. J. Appl. Physiol. 108, 1691–1700. 10.1152/japplphysiol.91539.2008 20185626

[B42] Sayed K. E. Macefield V. G. Hissen S. L. Joyner M. J. Taylor C. E. (2016). Rate of rise in diastolic blood pressure influences vascular sympathetic response to mental stress. J. Physiol. 24, 7465–7482. 10.1113/JP272963 PMC5157061 27690366

[B43] Schmidt H. D. Duman R. S. (2010). Peripheral BDNF produces antidepressant-like effects in cellular and behavioral models. Neuropsychopharmacology 35, 2378–2391. 10.1038/npp.2010.114 20686454 PMC2955759

[B44] Seifert T. Brassard P. Wissenberg M. Rasmussen P. Nordby P. Stallknecht B.. (2010). Endurance training enhances BDNF release from the human brain. Am. J. Physiol. Regul. Integr. Comp. Physiol. 298, R372–R377. 10.1152/ajpregu.00525.2009 19923361

[B45] Seifert T. Secher N. H. (2011). Sympathetic influence on cerebral blood flow and metabolism during exercise in humans. Prog. Neurobiol. 95, 406–426. 10.1016/j.pneurobio.2011.09.008 21963551

[B46] Sloand J. A. Hooper M. Izzo J. L. (1988). Effects of circulating norepinephrine on platelet, and red blood cell counts by alphal-adrenergic. Am. J. Cardiol. 63, 1140–1142. 10.1016/0002-9149(89)90096-9 2705387

[B47] Stewart I. B. Warburton D. E. Hodges A. N. H. Lyster D. M. McKenzie D. C. (2003). Cardiovascular and splenic responses to exercise in humans. J. Appl. Physiol. 94, 1619–1626. 10.1152/japplphysiol.00040.2002 12482773

[B48] Stratton J. R. Halter J. B. Hallstrom A. P. Caldwell J. H. Ritchie J. L. (1983). Comparative plasma catecholamine and hemodynamic responses to handgrip, cold pressor and supine bicycle exercise testing in normal subjects. J. Am. Coll. Cardiol. 2, 93–104. 10.1016/S0735-1097(83)80381-7 6853921

[B49] Szuhany K. L. Bugatti M. Otto M. W. (2015). A meta-analytic review of the effects of exercise on brain-derived neurotrophic factor. J. Psychiatr. Res. 60, 56–64. 10.1016/j.jpsychires.2014.10.003 25455510 PMC4314337

[B50] Tamura S. Suzuki H. Hirowatari Y. Hatase M. Nagasawa A. Matsuno K.. (2011). Release reaction of brain-derived neurotrophic factor (BDNF) through PAR1 activation and its two distinct pools in human platelets. Thromb. Res. 128, e55–e61. 10.1016/j.thromres.2011.06.002 21924459

[B51] Thoenen H. Huerlimann A. Haefely W. (1964). The effect of sympathetic nerve stimulation on volume, vascular resistance, and norepinephrine output in the isolated perfused spleen of the cat, and its modification by cocaine. J. Pharmacol. Exp. Ther. 143, 57–63. 14112308

[B52] Wadenvik H. Kutti J. (1988). The spleen and pooling of blood cells. Eur. J. Haematol. 41, 1–5. 10.1111/j.1600-0609.1988.tb00861.x 3042452

[B53] Walsh J. J. Scribbans T. D. Bentley R. F. Kellawan J. M. Gurd B. Tschakovsky M. E. (2016). Neurotrophic growth factor responses to lower body resistance training in older adults. Appl. Physiol. Nutr. Metab. 41, 315–323. 10.1139/apnm-2015-0410 26886517

[B54] Yarrow J. F. White L. J. McCoy S. C. Borst S. E. (2010). Training augments resistance exercise induced elevation of circulating brain derived neurotrophic factor (BDNF). Neurosci. Lett. 479, 161–165. 10.1016/j.neulet.2010.05.058 20553806

[B55] Ziegenhorn A. A. Schulte-Herbrüggen O. Danker-Hopfe H. Malbranc M. Hartung H.-D. Anders D.. (2007). Serum neurotrophins–a study on the time course and influencing factors in a large old age sample. Neurobiol. Aging 28, 1436–1445. 10.1016/j.neurobiolaging.2006.06.011 16879899

